# Asymmetric mandibular arch distalization with third-molar extractions for nonsurgical correction of skeletal Class III open bite and mandibular deviation: A case report

**DOI:** 10.1097/MD.0000000000045919

**Published:** 2025-11-14

**Authors:** Viet Anh Nguyen, Thi Minh Anh Ha, Hong Le Doan

**Affiliations:** a Faculty of Dentistry, Phenikaa University, Hanoi, Vietnam; b Private Practice, Viet Anh Orthodontic Clinic, Hanoi, Vietnam.

**Keywords:** anterior open bite, buccal-shelf miniscrews, mandibular distalization, skeletal Class III malocclusion, temporary anchorage device, third-molar extraction

## Abstract

**Rationale:**

This case report aimed to describe a premolar preservation, nonsurgical approach for adult skeletal Class III open bite using third-molar extraction and buccal-shelf miniscrew-anchored mandibular arch distalization.

**Patient concerns:**

A 27-year-old female presenting with a 2 mm anterior open bite, bilateral Class III molar and canine relationships, mild bimaxillary crowding, and midline deviations underwent extraction of all third molars.

**Diagnoses:**

Skeletal Class III malocclusion secondary to excessive mandibular growth in a horizontal growth pattern, Class III dental relationships with marked proclination of the maxillary and mandibular incisors, anterior crossbite and open bite, and leftward mandibular deviation.

**Interventions:**

Fixed appliances were bonded to initiate leveling and alignment. Two buccal-shelf miniscrews provided anchorage for en-masse mandibular distalization with power chains and Class III elastics. Interproximal enamel reduction refined the incisal morphology of both the maxillary and mandibular incisors.

**Outcomes:**

After 19 months of active treatment, all objectives were achieved. Bilateral Class I canine and molar relationships were established, overjet normalized from –1.4 mm to +2.8 mm, and overbite from –1.8 mm to +0.9 mm. The upper lip remained essentially stable, whereas roughly 2 mm of lower-lip retrusion permitted the upper lip to overlap the lower. Both arches were well leveled and aligned, with coincident dental and facial midlines.

**Lessons:**

Selective third-molar extraction combined with buccal-shelf miniscrew-anchored mandibular distalization is an effective nonsurgical alternative for adult skeletal Class III open-bite cases, preserving healthy premolars and delivering stable occlusal and esthetic outcomes.

## 1. Introduction

In adult populations, skeletal Class III malocclusion is among the least prevalent angle classifications, with a pooled global prevalence of 5.93 ± 4.69% (range 0.8–26.7 %).^[1]^ Regional variability is marked, with Southeast Asian cohorts exhibiting rates as high as 15.8 %.^[2]^ Anterior open bite likewise demonstrates wide epidemiologic spread (ranging from 1.6–47.1% across age groups and geographic regions) with a pooled prevalence of 16.5% (95% CI 12.3–21.2%) among children and adolescents.^[3]^

Jaw deformities arise from heterogeneous causes, including skeletal growth discrepancies, dentoalveolar compensations, congenital anomalies, and post-traumatic sequelae, each with distinct functional and aesthetic implications. Epidemiologic series of surgically managed mandibular fractures underscore trauma’s contribution to occlusal disturbances and asymmetry, with treatment pathways spanning open reduction and internal fixation to delayed orthognathic correction when residual deformity persists.^[4]^ Minor mandibular deviation can be managed nonsurgically when dental compensations and occlusal interferences are addressed.^[5,6]^

For nongrowing adults presenting with combined Class III/open-bite malocclusions, definitive correction is traditionally achieved via bimaxillary orthognathic surgery, which yields 70% to 100% long-term stability in systematic reviews.^[7]^ Orthodontic camouflage through first-premolar extractions can induce counterclockwise mandibular rotation and reduce the mandibular plane angle but frequently results in upper-lip flattening and accentuated lip retrusion.^[8]^ The advent of temporary anchorage devices has facilitated pure dentoalveolar compensation (whether by molar intrusion or arch distalization) offering a less invasive route with comparable occlusal improvements.^[9]^ However, literature on premolar preservation protocols that employ the extraction of only third molars with buccal-shelf miniscrew-anchored mandibular distalization remains sparse.

The objective of this case report is to describe the treatment rationale, biomechanical sequence, and clinical outcomes of a 27-year-old female with skeletal Class III malocclusion and a 3 mm anterior open bite, managed nonsurgically through selective third-molar extractions and miniscrew-supported mandibular arch distalization.

## 2. Case presentation

### 2.1. Diagnosis and etiology

A 27-year-old female presented to our orthodontic clinic with the chief complaint of inability of the incisors to contact. She had no relevant personal or family health issues.

**Figure 1.**
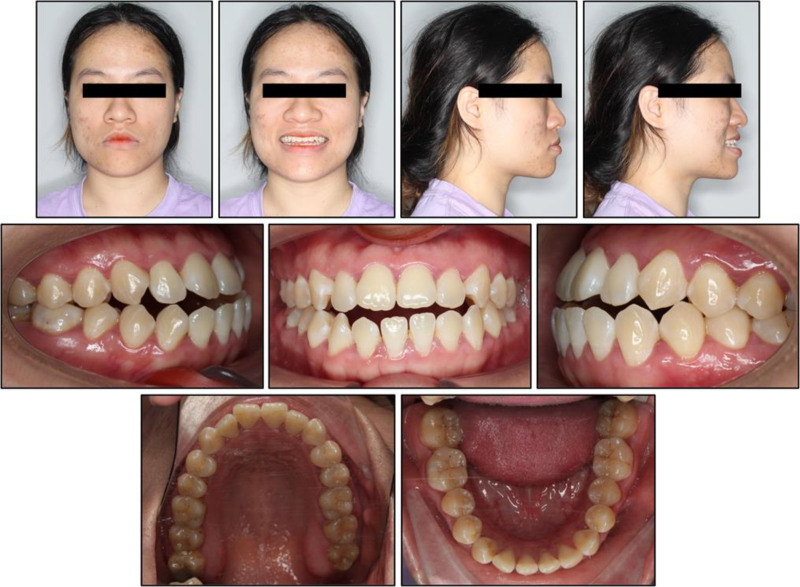
Pretreatment intraoral and extraoral photographs.

On frontal view, the patient demonstrated a balanced facial thirds and mild mandibular deviation 5 mm to the left of the facial midline (Fig. 1). Lateral assessment revealed a concave profile with a prognathic mandible and increased lower facial height; the lower lip and chin were prominent. Upon smiling, the maxillary incisors were not fully exposed, and the buccal corridors were reduced. There were no signs or symptoms of temporomandibular joint dysfunction.

Intraorally, she exhibited bilateral Class III canine and first molar relationships, more pronounced on the right side. Mild crowding was noted in the maxillary and mandibular anterior segment. The maxillary dental midline was shifted 1 mm to the right, while the mandibular dental midline was shifted 3 mm to the left. The maxillary incisors failed to overlap the mandibular incisors. No other dental or periodontal abnormalities were observed, aside from localized 1-mm gingival recession at the mandibular first molars at baseline (Table 1).

**Table 1:** Pre- and posttreatment periodontal summary.

Metric	Pretreatment	Posttreatment
Mean probing depth (mm)	1.9	1.9
Probing depth range (mm)	1–3	1–3
Sites with PD ≥ 4 mm (n, %)	0 (0%)	0 (0%)
Mean clinical attachment level (mm)	≈2.0	≈1.9
Max clinical attachment level (mm)	≤3	≤3
Full-mouth bleeding on probing (%)	6%	4%
Full-mouth plaque score (%)	18%	12%
Gingival margin (relative to cemento-enamel junction)	All 0 except mandibular first molars (−1) (recession)	All 0
Teeth with mobility ≥ 2 (n)	0	0
Furcation involvement ≥ II (n)	0	0

**Figure 2.**
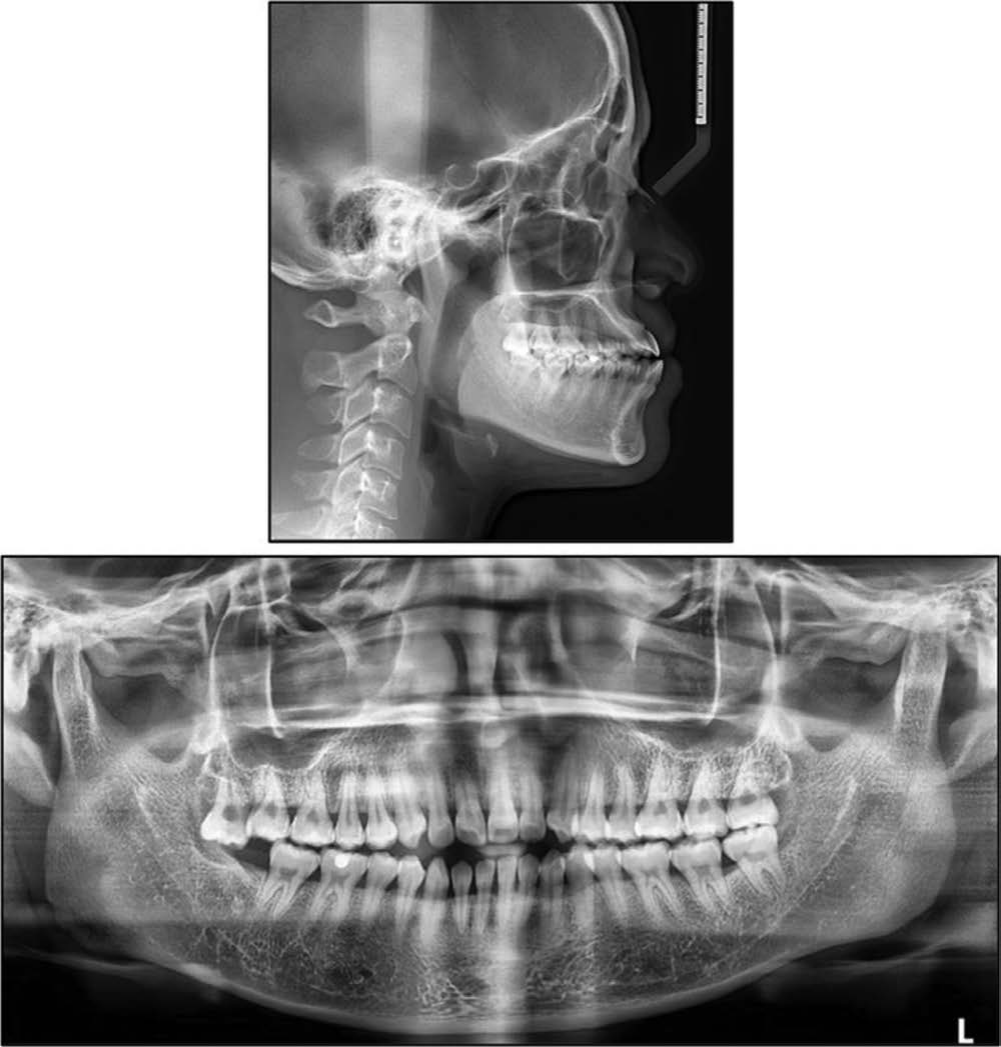
Pretreatment lateral cephalogram and panoramic radiograph.

A panoramic radiograph demonstrated retention of 3 third molars, with the mandibular right third molar having been previously extracted (Fig. 2). There was mild generalized horizontal and angular alveolar bone loss evident around most remaining teeth.

Pre-treatment lateral cephalometric analysis (Table 2) confirmed a pronounced skeletal Class III relationship, with a sella-nasion-point A angle of 80.36° and a sella-nasion-point B angle of 84.14°, resulting in a point A-nasion-point B angle of −3.78°. The Frankfort-mandibular plane angle was 16.28°, and the Wits appraisal measured −7.8 mm, both indicative of a horizontal growth pattern. Dentally, the maxillary incisors showed marked proclination (upper incisor to nasion-point A angle 39.77°/12.38 mm), while the mandibular incisors were similarly proclined (lower incisor to nasion-point B angle 34.9°/7.95 mm). The incisor-mandibular plane angle and the upper incisor to sella-nasion plane angle were 104.1° and 120.13°, respectively. Soft-tissue assessment demonstrated a nasolabial angle of 84.92° and retrusion of the upper and lower lips by 4.63 mm and 1.04 mm, respectively, behind the esthetic line. Negative overjet (−1.4 mm) and overbite (−1.8 mm) values confirmed the presence of an anterior open bite.

**Table 2:** Cephalometric mesurements.

Mesurements	Norm (mean ± SD)	Pretreatment	Posttreatment
Skeletal			
SNA (°)	81.08 ± 3.7	80.36	80.32
SNB (°)	79.17 ± 3.8	84.14	83.02
ANB (°)	2.46 ± 1.8	−3.78	-2.70
FMA (°)	25 ± 4.0	16.28	16.05
Wits (mm)	−0.33 ± 2.7	−7.80	−5.03*
Dental			
U1-NA (°, mm)	22.0 ± 5.0, 4.0 ± 3.0	39.77, 12.38	42.21, 13.26
L1-NB (°, mm)	25.0 ± 5.0, 4.0 ± 2.0	34.92, 7.95	23.87*, 6.39*
U1-SN (°)	105.28 ± 6.6	120.13	122.53
IMPA (°)	90 ± 3.5	104.14	94.12*
Overjet (mm)	2.0 ± 2.0	−1.37	2.82*
Overbite (mm)	2.0 ± 2.0	−1.80	0.87*
Soft-tissue			
Nasolabial angle (°)	95 ± 5	84.29	81.74
UL/E-line (mm)	0 ± 2.0	−4.63	−4.56
LL/E-line (mm)	0 ± 2.0	−1.04	−3.11*

ANB = point A-nasion-point B, E‐line = esthetic line, FMA = Frankfort-mandibular plane angle, IMPA = incisor-mandibular plane angle, L1-NB = lower incisor to nasion‐point B, SNA = sella-nasion-point A, SNB = sella-nasion-point B, U1-NA = upper incisor to nasion‐point A, U1-SN = upper incisor to sella-nasion plane.

* Denotes clinically meaningful change.

The diagnosis revealed a skeletal Class III malocclusion attributable to excessive mandibular growth with a predominantly horizontal growth pattern, with bilateral Class III dental relationships accompanied by marked proclination of both maxillary and mandibular incisors, anterior crossbite, and open bite, and leftward mandibular deviation.

### 2.2. Treatment objectives

The treatment objectives were first to correct the skeletal Class III relationship by addressing excessive mandibular growth and a horizontal growth pattern. Subsequently, bilateral Class I molar and canine relationships were to be established, and the 3 mm anterior open bite was closed to achieve a positive overjet and overbite. Concurrently, both arches were leveled and aligned to resolve mild maxillary anterior crowding, and the dental midlines were centered by correcting the 1 mm maxillary and 3 mm mandibular deviations. Furthermore, incisor inclinations were optimized to enhance occlusal function and the soft-tissue profile, all while preserving periodontal health through maintenance of alveolar bone support in regions of horizontal and angular bone loss.

### 2.3. Treatment alternatives

The treatment plan encompassed 3 distinct therapeutic approaches. The ideal treatment option was 2-arch alignment followed by orthognathic surgery. Bimaxillary orthognathic surgery offers definitive correction of the underlying skeletal discrepancy, reliably normalizing both occlusal relationships and facial balance.^[10]^ However, this single-stage bi-jaw procedure carries a significant risk of postoperative relapse and may leave residual occlusal or skeletal disharmonies when large jaw movements are performed.^[11]^ In addition, the extensive nature of bimaxillary surgery is associated with considerable intraoperative blood loss and prolonged anesthesia time, which in turn heightens the likelihood of surgical site infection, postoperative swelling, and neurosensory disturbances of the inferior alveolar and lingual nerves.^[12]^

The second alternative, including the extraction of the upper seconds and lower first premolar, offers a nonsurgical camouflage approach in hyperdivergent or anterior open-bite patients, since space closure mechanics can promote counterclockwise rotation of the occlusal plane and mandible, resulting in a measurable reduction of the mandibular plane angle.^[13]^ However, the extraction of first premolars inherently reduces the anterior dental arch volume, which in turn diminishes the structural support for perioral soft tissues and promotes retraction of the incisors. Both the upper and lower lips may retrude, leading to a notably flattened facial profile. This profile flattening can be especially pronounced in patients who present with thin lip morphology or require extensive anterior retraction, thereby potentially exacerbating concerns regarding lip fullness and overall facial harmony.^[14]^

Finally, by extracting only the third molars and employing distalization of the mandibular arch, this strategy preserves all sound premolars and obviates their removal. However, it requires insertion of skeletal anchorage devices. Although temporary anchorage minimizes patient compliance issues, placement of miniscrews is not without predictable side effects. Chronic soft-tissue inflammation and mucosal overgrowth at the insertion site have been reported in up to 85% of cases, and survey data indicate screw loosening may occur in nearly all devices if placement protocols are not meticulously followed.^[15,16]^ After a comprehensive review of each option’s advantages and limitations, the patient chose the third treatment strategy.

### 2.4. Treatment progress

Prior to orthodontic treatment, initial periodontal therapy was completed, including professional prophylaxis with site-specific scaling, root surface debridement, and oral-hygiene reinforcement, achieving low full-mouth plaque and bleeding indices. All third molars were then removed under local anesthesia. After 1 week of uneventful healing, fixed appliances were bonded. For aesthetic and financial considerations, 0.022 × 0.026-inch crystal ceramic SmartLine SL brackets (Medico, Korea) were placed on the maxillary dentition, while 0.022 × 0.026-inch self-ligating stainless-steel SmartLine SL brackets (Medico, Korea) were bonded to the mandibular arch.

**Figure 3.**
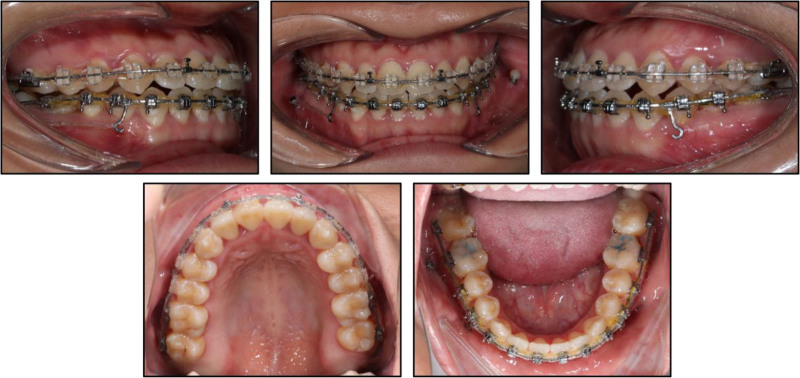
Intraoral photographs at 10 months into treatment. Distalization was discontinued on the left side and continued on the right to correct the right Class III relationship and translate the mandibular dental midline to the right.

**Figure 4.**
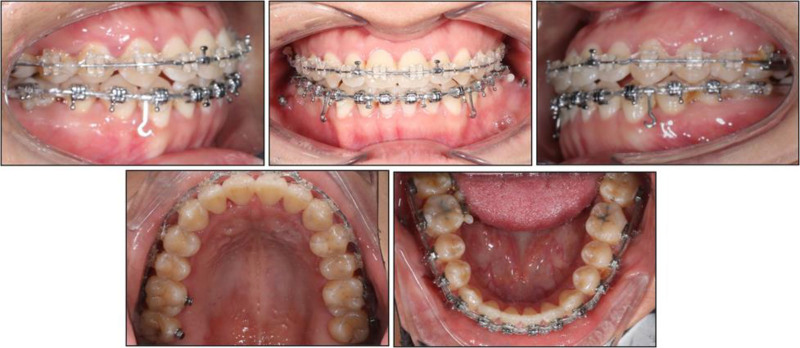
Intraoral photographs at 17 months into treatment.

Initial alignment began with a 0.014-inch nickel–titanium (NiTi) archwire. Archwire progression followed 0.016-inch, 0.016 × 0.022-inch, and 0.019 × 0.025-inch NiTi wires, finishing with a 0.018 × 0.025-inch stainless-steel wire. At this stage, two 1.8 × 10 mm miniscrews (Jeil Medical, Seoul, Korea) were placed in the mandibular buccal shelf (chosen for its thicker cortical bone) to serve as skeletal anchorage. Power chains were engaged from an interbracket hook between the canine and first premolar to each miniscrew. Given the mandibular dental midline deviation of 3 mm to the left, the patient wore right-sided Class III intermaxillary elastics (¼-inch, 3.5 oz) daily. Meanwhile, an asymmetric prescription was used, with heavier, longer-duration traction on the right (300 g) than on the left (150 g) to drive a rightward midline shift (Fig. 3). Interdisciplinary collaboration with restorative dentistry enabled careful interproximal enamel reduction in both arches to refine incisor contours and minimize black-triangle spaces. After 17 months, bilateral Class I molar and canine relationships were achieved, and both overjet and overbite were normalized. Ideal intercuspation resulted from counter-clockwise rotation of the mandible induced by distalization mechanics, coupled with controlled incisor extrusion and molar intrusion (Fig. 4).

A short finishing phase ensued, during which selected brackets were repositioned to correct minor rotations, and intermaxillary elastics were continued to perfect intercuspation. Patient compliance with daily elastic wear and meticulous oral hygiene instructions was critical to achieving these outcomes.

At 19 months, all appliances were removed. Fixed lingual retainers were bonded canine-to-canine in both arches, and clear thermoplastic retainers were delivered for full-time wear. Follow-up visits are scheduled every 6 months to monitor stability and detect any early signs of relapse.

### 2.5. Treatment results

**Figure 5.**
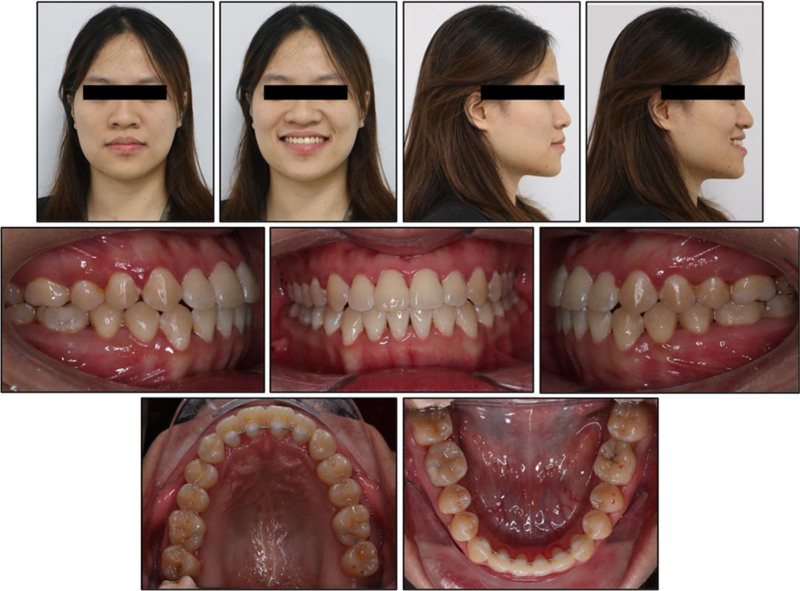
Posttreatment intraoral and extraoral photographs.

**Figure 6.**
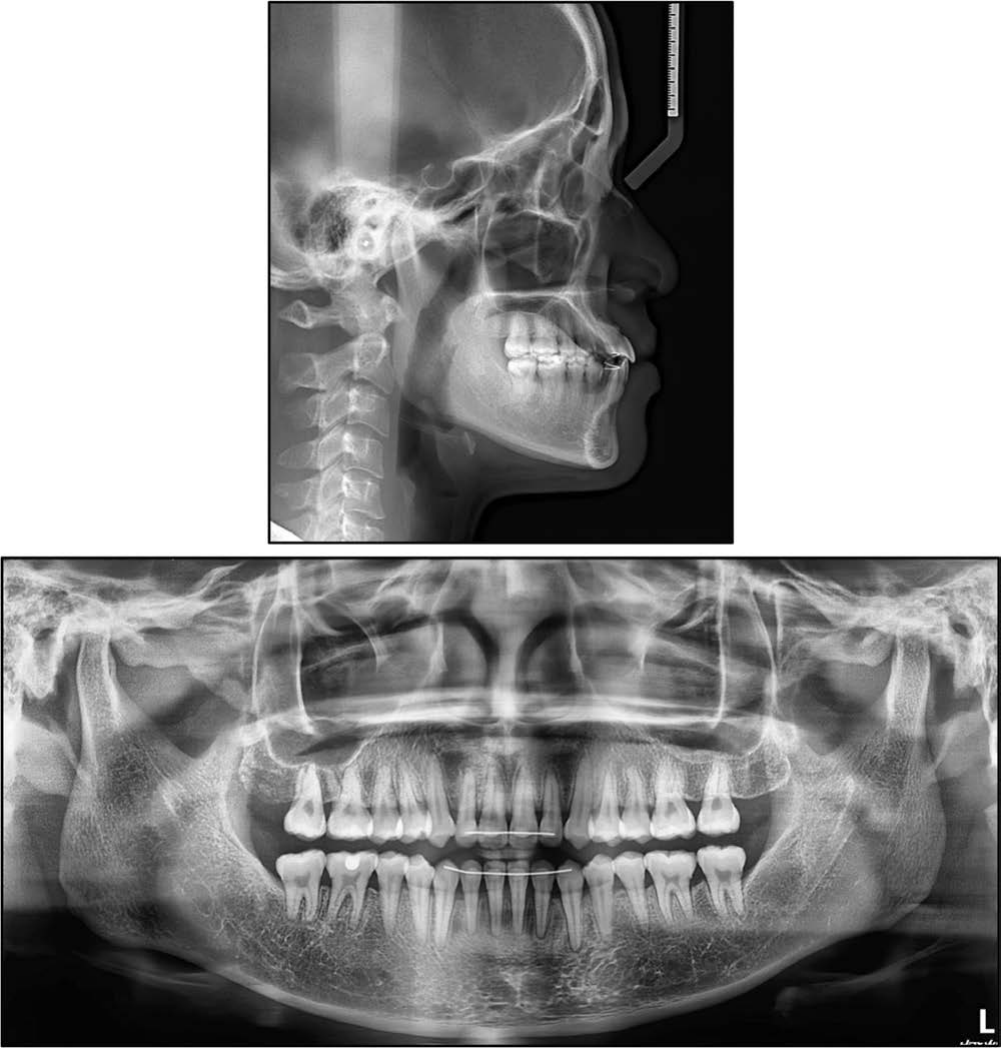
Posttreatment lateral cephalogram and panoramic radiograph.

Overall, posttreatment evaluation confirmed that all treatment objectives were met, with a stable, harmonious occlusion and enhanced facial esthetics. While menton deviation from the facial midline remained 5 mm pre- to posttreatment, mandibular dental midline shift improved from 3 mm to 0 mm. Both the maxillary and mandibular arches were well leveled and aligned; overjet and overbite values were normalized; bilateral Class I canine and molar relationships were established; ideal intercuspation was achieved without any posterior open bites or crossbites; and both dental midlines coincided precisely with the facial midline (Fig. 5). Panoramic radiography demonstrated maintained alveolar bone levels, parallel roots, and no signs of root resorption (Fig. 6). No radiographic worsening of periodontal support was observed at miniscrew sites, consistent with placement in extra-alveolar bone (buccal shelf). Periodontal status was maintained overall; the localized 1-mm gingival recession at the mandibular first molars diminished after treatment, plausibly aided by molar intrusion associated with buccal-shelf distalization.

Posttreatment cephalometric analysis (Table 2) demonstrated slight skeletal and marked dental improvements relative to the pretreatment values. Skeletally, sella-nasion-point A remained essentially unchanged at 80.32° (pretreatment 80.36°), indicating maintenance of maxillary position, while sella-nasion-point B slightly decreased from 84.14° to 83.02°, yielding an improved point A-nasion-point B of −2.70° (pretreatment −3.78°). The Wits appraisal improved from −7.80 mm to −5.03 mm, and the Frankfort-mandibular plane angle was slightly reduced to 16.05° (pretreatment 16.28°), indicating a slight counterclockwise autorotation of the mandible that possibly contributed to anterior open-bite correction.

**Figure 7.**
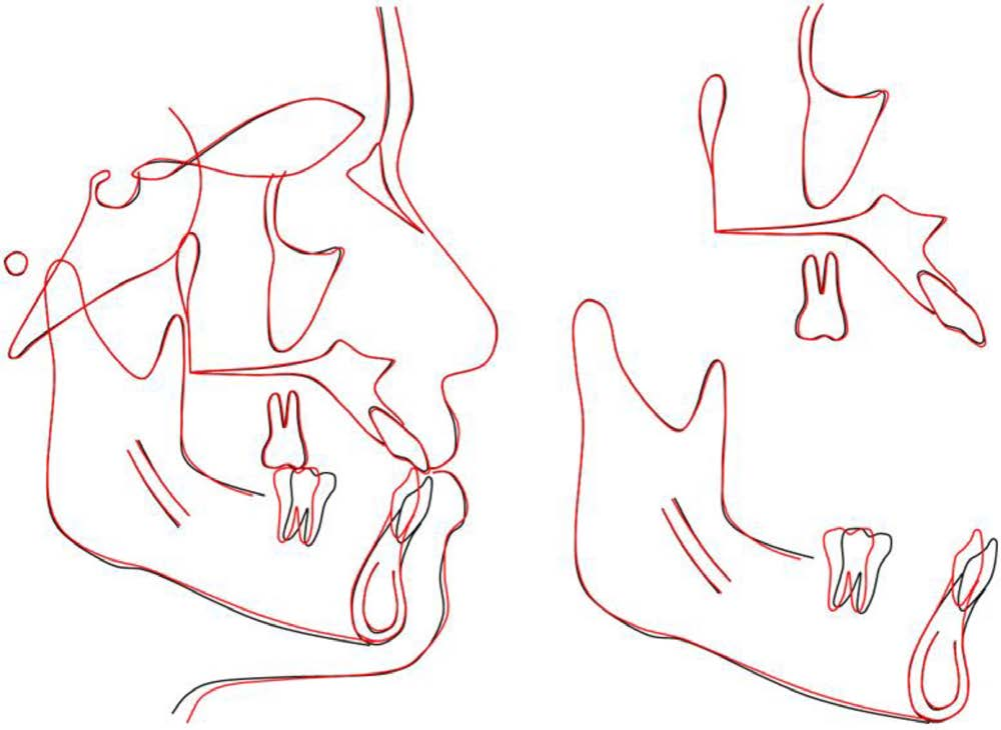
Overall and regional cephalometric superimpositions.

Dentally, maxillary incisor inclination further increased from U1–NA 39.77°/12.38 mm to 42.21°/13.26 mm, and U1–SN rose from 120.13° to 122.53°, reflecting continued labial movement to establish positive overjet. Mandibular incisors were uprighted significantly, with L1–NB reducing from 34.92°/7.95 mm to 23.87°/6.39 mm and incisor-mandibular plane angle decreasing from 104.14° to 94.12°, placing both within normative ranges. Overjet normalized from −1.37 mm to +2.82 mm, and overbite from −1.80 mm to +0.87 mm, confirming successful open-bite closure (Fig. 7).

Soft-tissue changes paralleled these dental corrections: the nasolabial angle increased slightly to 81.71° (pretreatment 84.29°). Upper-lip position remained essentially unchanged (pretreatment: −4.63 mm; posttreatment: −4.56 mm), whereas lower-lip retrusion increased from −1.04 mm to −3.11 mm, reflecting greater posterior displacement of the lower lip relative to the upper lip. Collectively, these cephalometric findings attest to effective skeletal camouflage, controlled vertical mechanics, and enhanced soft-tissue profile following nonsurgical distalization.

**Figure 8.**
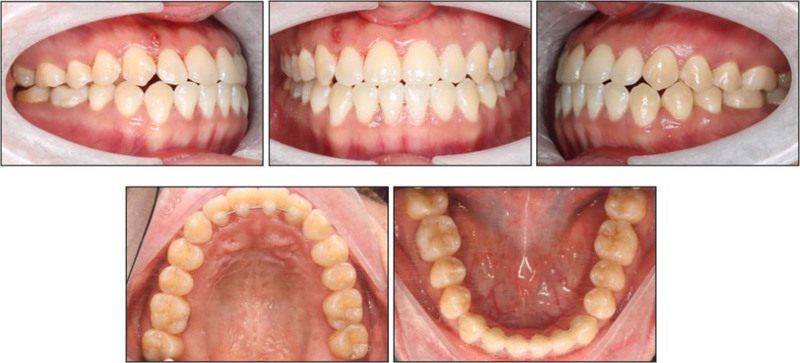
Two-year post-retention intraoral and extraoral photographs.

At 24-month follow-up, there was mild relapse of reduced overjet and overbite and a slight return toward Class III molar tendency, possibly influenced by macroglossia. However, alignment remained stable (Fig. 8).

## 3. Discussion

In this case, treatment was focused on targeted removal of only the third molars combined with biomechanically controlled mandibular distalization using buccal-shelf miniscrews, thereby preserving all healthy premolars and avoiding both orthognathic surgery and first-premolar extractions. Although previous reports have documented soft-tissue inflammation and mucosal overgrowth in up to 85% of miniscrew sites and a high incidence of screw loosening without strict protocol adherence, meticulous flap management and precise insertion technique in this patient minimized these complications, yielding rapid closure of the 3 mm anterior open bite, ideal intercuspation, and improved facial balance.^[15]^

Management options for skeletal Class III malocclusions comprised orthognathic surgery for skeletal correction, extraction-based orthodontics, and TAD-assisted dentoalveolar camouflage. Surgery offers definitive skeletal correction but entails operative morbidity; extraction strategies can reduce protrusion and crowding but may compromise posterior support; TAD camouflage leverages skeletal anchorage to reposition dentoalveolar units while minimizing extractions and surgery at the cost of potential hardware-related complications.

**Figure 9.**
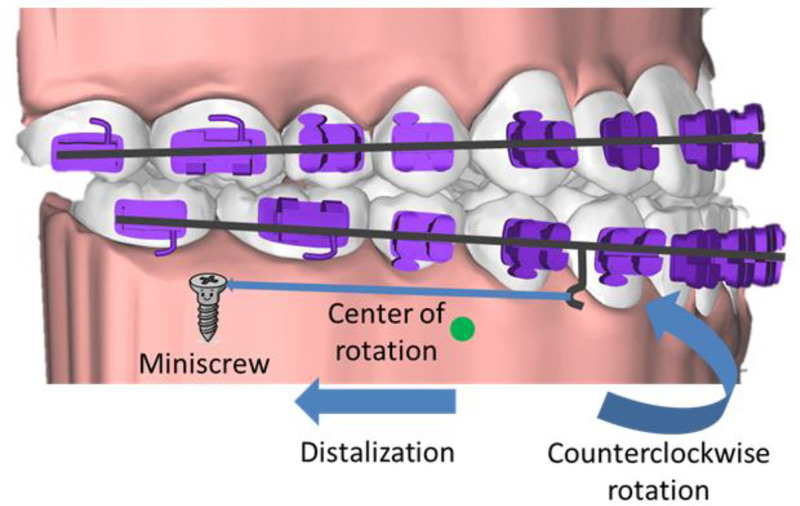
Mechanics of mandibular en-masse distalization with extra-alveolar buccal-shelf miniscrews.

From a biomechanical standpoint, en-masse distalization of the mandibular dentition via buccal-shelf miniscrew anchorage generates a resultant force vector applied apical to the dentoalveolar center of resistance, thereby imparting a counterclockwise pitching moment about the center of rotation of the mandibular dentoalveolar segment in the sagittal plane (Fig. 9).^[17]^ This force-couple induces mandibular autorotation driven by selective intrusion of the posterior dentoalveolar units (anchored by the miniscrews) and controlled extrusion of the anterior incisors, effecting a reduction in mandibular plane inclination, closure of the anterior vertical discrepancy, and anterior facial third advancement.^[18]^ The orchestrated intrusion–extrusion mechanics concurrently recontour the occlusal functional plane, optimize incisal guidance, and facilitate stable Class I intercuspation while preserving the lower facial height.^[19]^ Furthermore, targeted posterior crown intrusion prevents undesirable posterior extrusion and dentoalveolar flaring, reinforcing this modality’s efficacy in camouflaging Class III open-bite malocclusions.^[20]^ Practically, a deliberate right-side overactivation of the buccal-shelf distalization (greater force magnitude and longer moment arm on the right) generated an asymmetric yaw moment that translated the mandibular dental midline rightward-consistent with the observed correction toward coincidence.

By contrast, bimaxillary orthognathic surgery remains the most definitive modality for skeletal Class III correction, with 1-year stability rates reported as high as 82%^[21]^; however, it carries the burdens of general anesthesia, hospitalization, increased cost, and potential relapse.^[22,23]^ Extraction of first premolars can facilitate counterclockwise rotation of the mandible and a reduction in mandibular plane angle in hyperdivergent/open-bite cases, yet is often accompanied by an average 2 to 3 mm flattening of the upper-lip profile and worsened lip retrusion. Miniscrew-anchored distalization, on the other hand, achieves bodily molar movement of 1.3 to 6.4 mm with minimal anchorage loss, albeit over a longer treatment period and requiring vigilant management of anchorage mechanics.^[24]^

Several clinical reports have demonstrated that targeted removal of mandibular third molars combined with controlled distalization mechanics can effectively correct skeletal Class III open bites while preserving healthy premolars and avoiding orthognathic surgery. Tai et al^[25]^ described a 32-year-old patient who underwent extraction of all lower third molars followed by buccal-shelf miniscrew-anchored en-masse distalization, achieving 5.2 mm of bodily movement and stable Class I molar and canine relationships at 2-year follow-up. Chen et al^[26]^ similarly reported a 28-year-old treated with only third-molar extractions and skeletal anchorage, resulting in 4.5 mm of distalization, complete anterior open-bite closure within 12 months, and no relapse at 18 months. Ren et al^[27]^ contrasted this by using interradicular miniscrews after third-molar removal, attaining comparable occlusal outcomes over an 18-month active phase but with mild posterior tipping. Together, these cases corroborate the present report’s strategy, confirming that selective third-molar extraction plus mandibular arch distalization can reliably resolve Class III open bites and achieve stable, esthetic, and functional results.

Our results (anterior openbite closure with slight counterclockwise mandibular rotation and 24-month maintenance) align with systematic reviews indicating that posterior intrusion with skeletal anchorage effectively corrects anterior open bite in adults and shows moderate stability at follow-up. Meta-analyses report meaningful skeletal/dental improvements with TAD-assisted intrusion, with stability ranging from moderate to high depending on protocol and retention.^[28–30]^

Several limitations of this case report warrant consideration. First, as a single-patient investigation, the outcomes cannot be broadly generalized to all skeletal Class III open-bite malocclusions. Second, the absence of a control or comparison group prevents isolation of the specific contributions of third-molar extraction and miniscrew-anchored distalization from other treatment factors. Finally, facial soft tissue and radiographic assessment were limited to 2-dimensional cephalometric and panoramic imaging; incorporation of 3-dimensional modalities (e.g., 3D facial scan, cone beam computed tomography) would permit more precise evaluation of bone remodeling and soft-tissue adaptation.

## 4. Conclusions

In conclusion, selective extraction of mandibular third molars combined with buccal-shelf miniscrew-anchored distalization achieved all treatment goals in this adult skeletal Class III open-bite case. This premolar preservation protocol preserved healthy dentition, established stable bilateral Class I canine and molar relationships, normalized overjet and overbite, and enhanced facial esthetics, all with minimal morbidity. As such, this technique represents a viable nonsurgical alternative to orthognathic surgery or first-premolar extractions for similar cases; however, further long-term studies are necessary to confirm its broader applicability and stability.

## Author contributions

**Conceptualization:** Viet Anh Nguyen.

**Formal analysis:** Viet Anh Nguyen, Hong Le Doan.

**Investigation:** Thi Minh Anh Ha.

**Methodology:** Viet Anh Nguyen, Hong Le Doan.

**Project administration:** Viet Anh Nguyen.

**Resources:** Viet Anh Nguyen.

**Software:** Viet Anh Nguyen.

**Supervision:** Viet Anh Nguyen.

**Validation:** Viet Anh Nguyen.

**Visualization:** Viet Anh Nguyen.

**Writing – original draft:** Thi Minh Anh Ha.

**Writing – review & editing:** Viet Anh Nguyen.
